# Revealing the Dependency of Dye Adsorption and Photocatalytic Activity of ZnO Nanoparticles on Their Morphology and Defect States

**DOI:** 10.3390/nano13131998

**Published:** 2023-07-03

**Authors:** Yuri Hendrix, Erwan Rauwel, Keshav Nagpal, Ryma Haddad, Elias Estephan, Cédric Boissière, Protima Rauwel

**Affiliations:** 1 Institute of Forestry and Engineering Sciences, Estonian University of Life Sciences, 51014 Tartu, Estonia; yurihendrix@gmail.com (Y.H.); keshav.nagpal@student.emu.ee (K.N.); 2 Institute of Veterinary Medicine and Animal Sciences, Estonian University of Life Sciences, 51006 Tartu, Estonia; erwan.rauwel@emu.ee; 3 Laboratoire de Chimie de la Matière Condensée de Paris (LCMCP), Collège de France, CNRS, Sorbonne Université, 75005 Paris, France; ryma.haddad@sorbonne-universite.fr (R.H.); cedric.boissiere@upmc.fr (C.B.); 4 Laboratoire Bioinginirie et Nanoscience (LBN), University of Montpellier, 34193 Montpellier, France; elias.estephan@umontpellier.fr

**Keywords:** nanoparticles, ZnO, green photocatalysis, surface defects, ROS

## Abstract

ZnO is an effective photocatalyst applied to the degradation of organic dyes in aqueous media. In this study, the UV-light and sunlight-driven photocatalytic activities of ZnO nanoparticles are evaluated. A handheld Lovibond photometer was purposefully calibrated in order to monitor the dye removal in outdoor conditions. The effect of ZnO defect states, i.e., the presence of zinc and oxygen defects on the photocatalytic activity was probed for two types of dyes: fuchsin and methylene blue. Three morphologies of ZnO nanoparticles were deliberately selected, i.e., spherical, facetted and a mix of spherical and facetted, ascertained via transmission electron microscopy. Aqueous and non-aqueous sol-gel routes were applied to their synthesis in order to tailor their size, morphology and defect states. Raman spectroscopy demonstrated that the spherical nanoparticles contained a high amount of oxygen vacancies and zinc interstitials. Photoluminescence spectroscopy revealed that the facetted nanoparticles harbored zinc vacancies in addition to oxygen vacancies. A mechanism for dye degradation based on the possible surface defects in facetted nanoparticles is proposed in this work. The reusability of these nanoparticles for five cycles of dye degradation was also analyzed. More specifically, facetted ZnO nanoparticles tend to exhibit higher efficiencies and reusability than spherical nanoparticles.

## 1. Introduction

Removal of toxic chemicals, such as dyes from industrial effluents requires innovative treatment methods. Dyes can be toxic and carcinogenic in high accumulated doses in the body [1,2,3,4]. Furthermore, other than the dye itself, molecular side-groups, e.g., toluene generated from the dyes during natural degradation processes are also harmful compounds [5]. In that regard, dye degradation in the presence of a photocatalyst appears to be an efficient and eco-friendly method that produces simple compounds, such as water and carbon dioxide. Photocatalytic nanomaterials such as TiO_2_ and ZnO are capable of fully degrading a large variety of organic and some inorganic molecules [6,7,8,9,10,11,12,13]. The dye-degradation mechanism involves electron transfer from the nanoparticle to the oxygen species, biomolecules or other organic compounds in its vicinity. While some concerns about the toxicity of semiconductor nanoparticles due to the creation of reactive oxygen species (ROS) have emerged [14,15,16,17], ZnO nanoparticles tend to be relatively safe, presenting cytotoxicity only in large quantities on inhalation and skin exposure [14,15,16]. In places with an abundance of natural sunlight, photocatalysis has shown to be an excellent option in terms of cost-effectiveness and sustainability. However, photocatalytic treatment of wastewater still needs further research in areas such as the degradation rates of different molecules, the influence of solar irradiation and their reusability. More particularly, the effect of dye adsorption by the nanoparticles on degradation efficiency is still ambiguous.

ZnO is a wide-bandgap semiconductor with a direct bandgap of 3.37 eV and is considered a highly efficient photocatalyst for wastewater treatment [7,8]. Absorption of light in semiconductors leads to the creation of excitons. The excited electron–hole pairs react with hydroxyl and oxygen molecules in their vicinity to form ROS that degrade organic molecules, such as dyes. In wide-bandgap semiconductors, the longer recombination lifetimes of excitons, augments the probability of ROS production through electron transfer. However, a wide bandgap also implies photo-absorption in the UV region, which in turn, renders them unsuitable for visible-light photocatalysis.

Previous reports have demonstrated that the ZnO-nanoparticle synthesis route has an enormous impact on its photocatalytic activity [7,8,18,19,20,21,22,23,24,25]. Synthesis routes tailor the morphology, size and specific surface of the nanoparticle. Besides the shape, the photocatalytic activity also depends on the crystalline quality of the material or the presence of defect states [18,19,20,21,25]. Since the lifetime of excitons is short (~322 ps) for bulk ZnO, only those able to reach the surface contribute to the photocatalytic activity through the creation of ROS [26]. Furthermore, defects in the material, such as oxygen (V_O_) and zinc vacancies (V_Zn_) act as traps and prolong the lifetime of the excitons [27]. Nevertheless, only defects on the surface of the nanoparticle participate in photocatalysis. On the other hand, volume-related defects in ZnO act as traps and recombination centers and prevent the photogenerated electron from reaching the surface. Therefore, the reduction of defects in general has been of interest for several photocatalytic water-splitting applications using oxynitride materials [28,29]. This was achieved through the increase in surface area of the photocatalysts. Other strategies for more efficient photocatalytic water splitting include adding a metal co-catalyst that acts as an electron trap and discourages radiative recombinations [30]. Nevertheless, in small nanoparticles, surface defects are dominant due to the high surface-to-volume ratio, while in larger nanoparticles, both surface and volume defects are present. Surface defects can also adsorb oxide radicals and other molecules [18,19]. In particular, V_O_ on the surface of the nanoparticles tends to capture excitons that are easily transferred to the organic molecules adsorbed by them; therefore, these defect sites act as active centers for dye degradation. 

In this work, we investigate the influence of the synthesis routes on the photocatalytic activity of ZnO nanoparticles through their size and morphology. Photocatalytic activity is known to be dependent on the morphology, nanoparticle size and defect states of ZnO. The adsorption and degradation of basic fuchsin (C_19_H_17_N_3_ · HCl) and methylene blue (C_16_H_18_ClN_3_S) by the ZnO nanoparticles are evaluated. For this purpose, the Lovibond handheld photometer, especially suited to on-site analysis (Lovibond photometer MD 600), was calibrated for both dyes. The aim was to devise an on-site technique for facile and rapid dye-concentration analyses. Besides experiments under UV-light at the lab-scale, the samples were also exposed to direct sunlight in order to assess their photocatalytic activity in outdoor conditions. In principle, a photocatalyst should be reusable for multiple cycles of dye degradation. Therefore, the reusability of the nanoparticles for several cycles of dye degradation is an important parameter to monitor in order to ensure sustainability, which is also addressed in this work. All in all, this study evaluates the ability of ZnO nanoparticles synthesized via three different routes to remove dyes with optimum turnover rates in a sustainable and practical way.

## 2. Methods

### 2.1. Materials and Reagents

Zinc acetate Zn(CH_3_CO_2_)_2_ (99.99%, Aldrich, St. Louis, MO, USA), benzyl amine (≥99.0%, Aldrich), zinc acetate anhydrous (99.9%, Aldrich), absolute ethanol (≥99.5%, Honeywell, Charlotte, NC, USA), NaOH (99.9%, Aldrich).

### 2.2. Synthesis of ZnO Nanoparticles

#### 2.2.1. Synthesis of ZnO A

Inside a glovebox (>1 ppm H_2_O), 3.41 mmol (626 mg) zinc acetate Zn(CH_3_CO_2_)_2_ (99.99%, Aldrich) was added to 20 mL benzyl amine (≥99.0%, Aldrich). The reaction mixture was transferred to a stainless-steel autoclave and carefully sealed. Thereafter, the autoclave was taken out of the glovebox and heated in a furnace at 300 °C for 2 days. The resulting milky suspension was centrifuged; the precipitate was thoroughly washed with ethanol and dichloromethane and subsequently dried in air at 60 °C. 

#### 2.2.2. Synthesis of ZnO B

For the synthesis of ZnO B nanoparticles, 183.48 mg zinc acetate anhydrous (99.9%, Aldrich) was dissolved in 20 mL of absolute ethanol (≥99.5%, Honeywell). The solution was maintained under continuous magnetic stirring at 65 °C until the precursor was fully dissolved. Later, 0.125 M of NaOH (99.9%, Aldrich) in 20 mL absolute ethanol was added dropwise to obtain a 1:2.5 molar ratio. Thereafter, the mixture was maintained at 65 °C under continuous magnetic stirring for 2 h. The resulting dispersion was centrifuged at 4500 rpm for 6 min and dried for 24 h at 60 °C in air.

#### 2.2.3. Synthesis of ZnO C

Firstly, 219.5 mg of zinc acetate dihydrate was dissolved in 20 mL of aqueous ethanol (70%). Then, the solution was maintained at 60 °C with a water bath under continuous magnetic stirring until a transparent solution was obtained. An amount of 0.125 M of NaOH (99.9%, Aldrich) in 20 mL aqueous ethanol (70%) was added drop-wise to obtain a 1:2.5 molar ratio. The aqueous ethanol was made with absolute ethanol (≥99.5%, Honeywell) and distilled water. The solution was maintained at 60 °C for another 2 h. The resulting dispersion was centrifuged and dried in air at 60 °C. 

### 2.3. Characterization

X-ray diffraction patterns were collected in Bragg–Brentano geometry using a Bruker D8 Discover diffractometer (Bruker AXS, Karlsruhe, Germany) with CuKα1 radiation (λ = 0.15406 nm) selected with a Ge (111) monochromator and LynxEye detector. Transmission electron microscopy (TEM) was carried out on a Tecnai G2 F20 equipped with a 200 kV field emission gun (FEG) and a Jeol 200Cx equipped with a LaB_6_ emitter. High-resolution TEM provides a point-to-point resolution of ~2.4 Å. Characteristics such as specific surface and porosity were measured during N_2_ adsorption–desorption experiments using a surface area and pore size analyzer (BELSORP mini2, Osaka, Japan). Samples were degassed overnight at a temperature of 110 °C. The specific surface and pore size distribution were determined from the linear part of the Brunauer–Emmet–Teller (BET) equation limited by Rouquerol’s rules in a range of relative pressures p/p_0_ from 0.05 to 0.35. The Barrett–Joyner–Halenda (BHJ) method was used on the desorption branch of the isotherm for calculating the pore size distribution. The optical absorbance of the ZnO samples was determined using an UV-Vis UV-1600PC spectrophotometer in the 300–700 nm region. The bandgap of the ZnO samples was subsequently calculated with Tauc plots. Photoluminescence (PL) spectroscopy was carried out on ZnO powders at room temperature with an excitation wavelength of 365 nm of a LSM-365A LED (Ocean insight, Orlando, FL, USA) with a specified output power of 10 mW. The emission was collected using a FLAME UV-Vis spectrometer (Ocean optics, Orlando, FL, USA) with spectral resolution of 1.34 nm. Raman spectra were collected using a WITec Confocal Raman Microscope System alpha 300R (WITec Inc., Ulm, Germany). Excitation in confocal Raman microscopy was generated using a frequency-doubled Nd:YAG laser (Newport, Irvine, CA, USA) at wavelengths of 532 nm and 633 nm with 50 mW maximum laser output power in a single longitudinal mode. The incident laser beam was focused onto the sample through a 20× NIKON (Nikon, Tokyo, Japan). The acquisition time and accumulation time for a single spectrum were set to 10 s.

### 2.4. Photocatalytic Degradation

The photocatalytic degradation of two different dyes, i.e., basic fuchsin (general-purpose grade, Fisher Chemical, Hampton, NH, USA) and methylene blue (high purity, biological stain, Thermos Scientific, Waltham, MA, USA) were investigated. Three different configurations were tested. The measurements were performed with a handheld Lovibond photometer (MD 600) which was calibrated against the UV-Vis spectrophotometer (UV-1600PC) as shown in the supporting data Figure S1. The dye removal was tracked by measuring the absorbance at or close to the main absorption peaks (i.e., 560 nm for basic fuchsin and 660 nm for methylene blue) at regular intervals for 3 h. The amount of residual dye in the solution is directly proportional to the absorbance. The removed amount can be calculated from the difference in the initial and residual dye concentrations taking into account the mass of the catalyst and the volume of the solution taken. This removal can be due to adsorption of the dye molecules onto the nanoparticles, as well as degradation of the molecules. The remaining dye concentration can be extrapolated from the calibration curve of Figure S1 as the absorbance is directly proportional to the concentration of the dye following Beers–Lambert law.

#### 2.4.1. Photocatalytic Activity under UV-Light 

For experiments in laboratory, 1 mg of each ZnO sample and 10 mL of 5 ppm dye solution were added to a Petri dish and placed under a UV-lamp of 365 nm excitation, consisting of four 9 W bulbs. The emission spectrum of the UV-lamp is provided in Figure S2 of the supporting information. To evaluate the reusability of the samples, the same experiment was repeated four times for each sample with a new solution.

#### 2.4.2. Photocatalytic Activity under Sunlight 

The second configuration was used to test the sample under solar irradiation in open air, on the roof of the Tehnikainstituut, Kreutzwaldi 56/1, Tartu, Estonia. For this, 1 mg of ZnO nanoparticles was mixed with a 10 mL aqueous solution containing 5 ppm of dye in a Petri dish. The irradiance and UV-index of the corresponding days and location are shown in Figure S3. All the experiments were performed on days with UV-index between 4–5. In order to counteract the evaporation of the solvent, a constant volume of 10 mL was maintained by adding distilled water before each absorbance measurement. 

#### 2.4.3. Adsorption of Dye and Its Influence on the Photocatalytic Activity under UV-Light

The third configuration enabled distinguishing the type of dye-removal process, i.e., photocatalytic degradation or adsorption. It also enabled evaluation of the influence of the adsorbed dye on the photocatalytic activity under UV light. The decrease in dye concentration after mixing 1 mg of ZnO nanoparticles with a 10 mL aqueous solution containing 5 ppm of dye in a Petri dish was monitored at several intervals. Photocatalysis was performed on these samples, first, immediately after attaining adsorption equilibrium and second, after 24 h of adsorption. 

**Figure 1:**
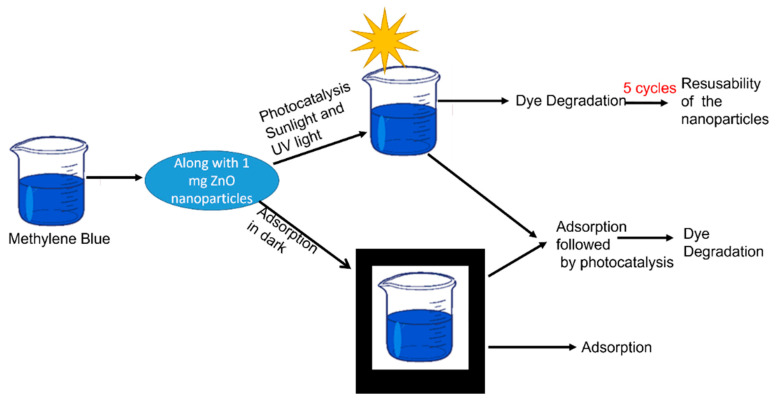
Procedure followed for evaluating the adsorption, photocatalytic efficiency and adsorption plus photocatalytic efficiency of ZnO A, ZnO B and ZnO C.

In order to elucidate the mechanisms involved in photocatalysis after dye adsorption, further experiments were carried out. After reaching an adsorption equilibrium, the nanoparticles were isolated from the dye solution via centrifugation. They were then exposed for three hours to the UV lamp, after which they were returned to the dye solution to check their reusability. This procedure was repeated 4 times. The procedure adopted to evaluate the adsorption and photocatalytic activity of the ZnO nanoparticles is provided in Figure 1.

## 3. Results

### 3.1. Crystal Structure and Morphology

**Figure 2:**
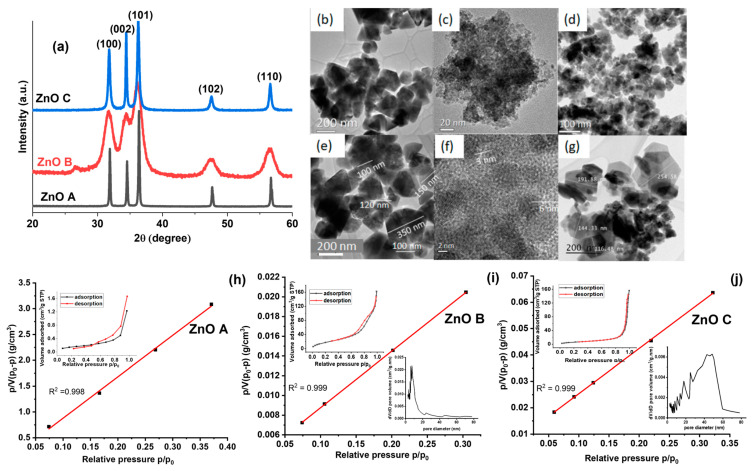
(**a**) X-ray diffraction patterns of the ZnO nanoparticles (JCPDS, card number 36-1451). TEM images of (**b**,**e**) ZnO A, (**c**,**f**) ZnO B, (**d**,**g**) ZnO C. (**h**–**j**) BET plots for N_2_ adsorption on ZnO A, ZnO B and ZnO C. Insets are the full adsorption isotherms for the three samples, along with their pore size distributions.

Diffractograms of the ZnO samples are shown in Figure 2a. The peaks (100), (002), (101), (102), and (110) correspond to the hexagonal Wurtzite structure (a = 3.25 Å and c = 5.20 Å) of ZnO (JCPDS, Card Number 36-1451). No secondary phases are visible in the XRD patterns, indicating that only single-phase ZnO nanoparticles were precipitated. The peak for ZnO B at 26° belongs to unreacted zinc acetate precursor [31].

The TEM images in Figure 2b–g provide an overview of the particle sizes and morphologies of the three ZnO samples. All three samples present different morphologies and particle sizes, due to differences in synthesis conditions (i.e., solvent, precursor, synthesis temperature and time). Among the three samples, ZnO A in Figure 2b displays the largest particle sizes with sharp edges and facets in hexagonal and triangular nanoparticles. The higher magnification image of ZnO A in Figure 2e consists of hexagonal and triangular-facetted nanoparticles. On the other hand, ZnO B, shown in Figure 2c, consists of much smaller agglomerates of nanoparticles that are spherical, exhibiting a porous structure. The HRTEM image of ZnO B in Figure 2f, illustrates spherical nanoparticles with an average size of 5 nm. The size of ZnO C nanoparticles is intermediate to the other two samples and nanoparticles with both sharp and smooth edges are visible in Figure 2d. Figure 2g is a higher magnification image of ZnO C, where several large hexagonally facetted nanoparticles are visible, along with less defined and smaller nanoparticles. The specific surface of the nanoparticles was calculated using the average size of the nanoparticles provided in the size distribution histograms in Figure S4. ZnO A, ZnO B and ZnO C have average particles sizes of 112 nm, 5 nm and 23 nm, respectively [20,21,31]. The hexagonally shaped nanoparticles were approximated to a sphere for specific surface calculations. Subsequently, the number of nanoparticles in 1 mg of the catalyst loading was calculated after determining the volume of the sphere, which was multiplied by its surface area. The theoretical specific surfaces of ZnO A, ZnO B and ZnO C are therefore 9.56 m^2^/g, 214 m^2^/g and 22.7 m^2^/g, respectively. Subsequently, the surface areas of dry powders were deduced from N_2_ physisorption using the BET method shown in Figure 2h–j [32]. The insets are the N_2_ physisorption isotherms. In particular, the isotherm of ZnO A is poorly resolved due to its very low surface area, even though we used more than one gram of powder, suggesting that the detection limit of the apparatus was reached. Sample ZnO A presents a type II adsorption isotherm characteristic of macroporous materials. Sample ZnO B presents an isotherm with a hysteresis loop in between type IV-H2 and IV-H3 characteristics of the presence of mesopores, probably due to the packing of the small ZnO particles observed in the TEM image of Figure 2. Finally, sample ZnO C presents an isotherm with a hysteresis loop above 0.9 in p/p_0_ of type IV-H3 which can be attributed to mesopores with slit-shaped porosities. Parameters such as the specific surface (S_BET_), BET constant (C_BET_), BET particle size (d_BET_), monolayer adsorption volume (V_m_) and pore volume (V_p_) are tabulated in Table 1. ZnO B presented the largest specific surface of 72 m²/g and the specific surface of ZnO C of 24 m²/g. In addition, the volume of the monolayer adsorption decreased with increasing particle size, owing to the decrease in the specific surface. The pore volume (V_p_) of samples ZnO B and C are approximately equal. Furthermore, ZnO B displayed a clear mesoporosity compared to ZnO C with a pore size distribution around 10 nm (inset of Figure 2i). For ZnO C, the average pore size distribution is higher at ~46 nm with minimum and maximum pore sizes of 10 nm and 60 nm (inset of Figure 2j), respectively. On the other hand, ZnO A displayed near-zero mesoporosity, suggesting that ZnO A consisted of large particles and that their packing creates a porosity larger than 50 nm and could not be evaluated by the present method. We noticed that for ZnO A, the presence of facetted and sharp-edged nanoparticles, could also promote their tight packing in dry-powder form. Our study however consisted of employing a colloidal dispersion of the nanoparticles in an aqueous dye solution that promotes the homogeneous distribution of nanoparticles without agglomeration. Therefore, the specific surfaces of the dried powders obtained from BET may not be entirely relevant for this present study, considering the fact that we are evaluating the photocatalytic activities of ZnO nanoparticles as colloidal suspensions in aqueous dyes.

ZnO B was prepared with absolute ethanol and an anhydrous zinc acetate precursor. On the other hand, ZnO C was prepared with aqueous ethanol and a dihydrate acetate precursor. Thus, the difference between these two synthesis methods is the presence of water molecules in the solvent and precursor for ZnO C. These water molecules increase the reaction rate of the hydrothermal synthesis route and in turn, engender the precipitation of larger nanoparticles [21]. On the other hand, ZnO A, which was synthesized via non-aqueous sol-gel routes using benzyl amine as a solvent was devoid of water. The characteristics of these nanoparticles are tabulated in Table 2. Therefore, the type of solvent plays a crucial role in the morphology of the synthesized nanoparticles and influences other properties, as given below.

### 3.2. Optical Characteristics

**Figure 3:**
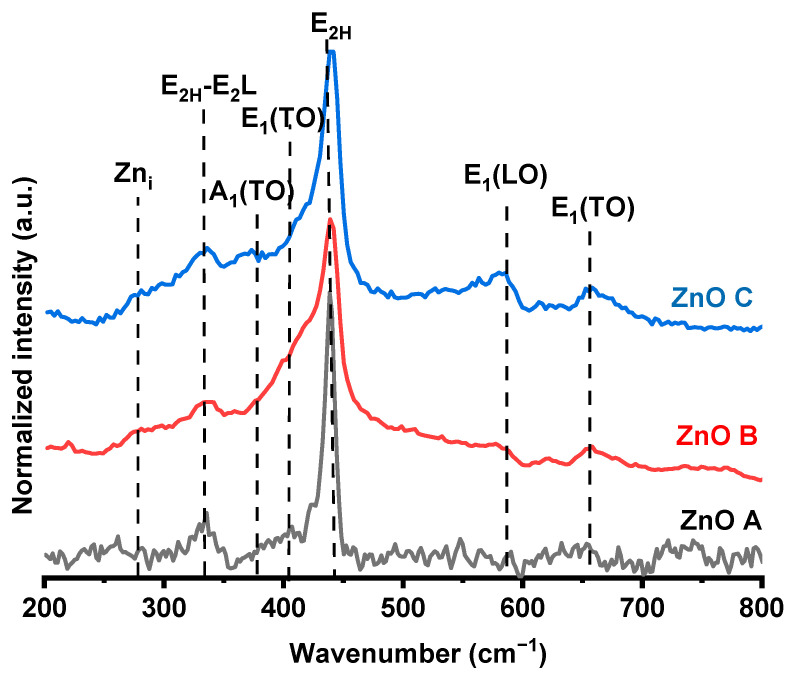
Normalized Raman spectra of the ZnO samples in the range of 100–800 cm^−1^.

The Raman spectra for the samples were acquired within the range of 200 cm^−1^ and 800 cm^−1^ in Figure 3. For the Samples ZnO B and ZnO C, the laser excitation was 532 nm. On the other hand, due to the high fluorescence exhibited by ZnO A under the green laser excitation, a red laser with an excitation wavelength of 633 nm was used. In fact, 532 nm corresponds to the defect level absorption in ZnO, which produced a fluorescence signal that in turn, dissimulated the Raman signal. Starting from lower frequencies, Raman signatures under 300 cm^−1^ are assigned to the vibrations of Zn_i_, and those above 300 cm^−1^ are assigned to the vibrations of oxygen atoms [33]. The peak at 275 cm^−1^ has been attributed to Zn_i_ or Zn_i_ clustering and is only visible in samples ZnO B and ZnO C [34,35]. The peak visible at ~320 cm^−1^ is a multiphonon scattering mode of E_2H_–E_2L_ and is related to the crystalline quality of the sample. In fact, E_2L_ involves the vibration of the heavy Zn sublattice [36], and the E_2H_ at 440 cm^−1^ corresponds to lattice–oxygen vibrations of Wurtzite ZnO. The relative intensity of this peak is the highest for ZnO A, owing to the large particle size. The other phonon modes obtained at ~585 cm^−1^ and ~667 cm^−1^ correspond to E_1_(LO) and E_1_(TO) modes, respectively [37]. In general, the E_2H_, E_2H_–E_2_L, E_1_(LO) modes involve the oxygen component of ZnO; especially, E_1_(LO) corresponds to oxygen-related defects [38]. For ZnO C, the E_2H_ mode has shifted to higher wave numbers, indicating a more stable lattice–oxygen configuration, or a lower amount of V_O_. On the other hand, the E_2H_ peak for the other two samples has shifted to slightly lower wavenumbers, indicative of a higher number of V_O_ [31]. The A_1_(TO) with E_1_(TO) modes reveal variations in lattice bonds affected by local changes induced by intrinsic defects. The E_1_(LO) mode is usually dominant in doped ZnO samples, where Zn is replaced by a metallic dopant that modifies the local charge distribution owing to the presence of V_Zn_ or Zn_i_ [39].

**Figure 4:**
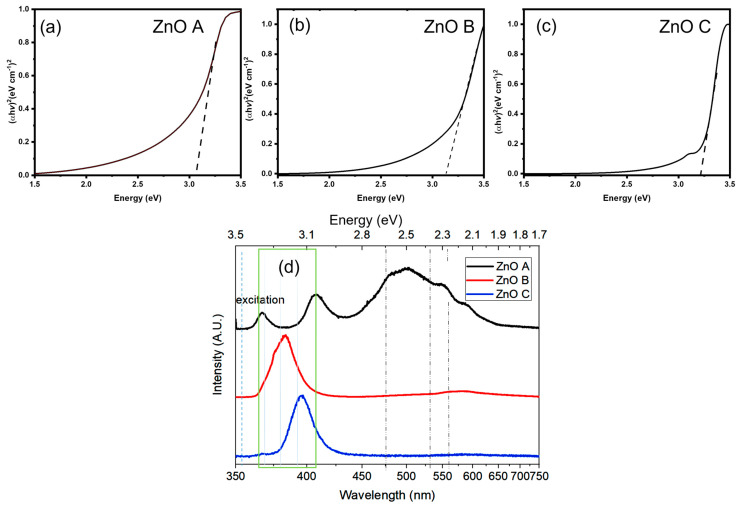
Tauc plots of (**a**) ZnO A, (**b**) ZnO B and (**c**) ZnO C and (**d**) photoluminescence emission spectra of ZnO A, B and C under 365 nm excitation.

UV-Vis absorption spectroscopy was carried out followed by Tauc plots (Shown in Figure 4) in order to determine the absorbance range and band gaps of ZnO nanoparticles. For all the samples, the band gap lies between 3.07 eV and 3.26 eV, which is typical for ZnO. For ZnO B and ZnO C (Figure 4b,c), the band tail tapers off at ~1.75 eV and 2 eV in the visible region, respectively. Variations in the band gaps for ZnO nanoparticles are likely due to the differences in the synthesis conditions [20,40]. In addition, errors in the band gap estimation from Tauc plots may be due to the presence of organic moieties as a result of the synthesis routes [41]. In general, Tauc plot allows accurate estimation of band gaps for bare semiconductors. 

Photoluminescence emission spectroscopy is necessary to understand the various radiative and non-radiative recombination mechanisms that could hinder or promote the photocatalytic activity of ZnO. The emission properties of the three ZnO samples are presented in Figure 4d. These emissions correspond to typical emission bands of ZnO with variations in intensities of certain emission signatures. In general, the ZnO emission spectra consist of two distinct bands called the near-band emission (NBE) and the defect level emission (DLE). NBE is in the UV region corresponds to band-to-band transitions [42]; DLE in the visible region arises from transitions between the conduction band or donor states to defect or band gap states. These defect states correspond to V_O_, Zn_i_, and V_Zn_ in these samples. ZnO emissions at ~2.5 eV and 2.2 eV correspond to volume (V_O_^+^) and surface (V_O_^++^) oxygen vacancy components, respectively, while the peak at ~3 eV corresponds to either Zn_i_ or V_Zn_ [20]. In the Raman spectrum of ZnO A in Figure 3, the vibrations corresponding to Zn_i_ are absent. In addition, in a previous study it was demonstrated that this sample was Zn-deficient and therefore, the emission at 3 eV is mostly likely due to V_Zn_ [43]. For ZnO B and C, redshifts of the NBE are due to shallow donor states, confirmed by the presence of Zn_i_ in their Raman spectra.

The ratio of the NBE to DLE is an indicator of the crystalline quality of ZnO. Therefore, ZnO C, with the highest ratio, exhibits the best crystalline quality among the three samples. Similarly, ZnO B displays a negligible DLE, which consists of mostly surface-related V_O_^++^. Despite the fact that the solvent and precursors used in the synthesis of ZnO B were non-aqueous and anhydrous, the presence of NaOH in the reaction serves as an oxygen supplying source via a hydrolytic reaction, which can reduce the quantity of V_O_^+^/V_O_^++.^ For ZnO A, the volume-related V_O_^+^ emission at 2.5 eV dominates the PL spectrum followed by the V_O_^++^ emission. Therefore, the presence of both types of oxygen vacancies can be correlated to the non-aqueous and non-hydrolytic synthesis route creating an oxygen-deficient synthesis environment.

### 3.3. Photocatalytic Dyedegradation 

**Figure 5:**
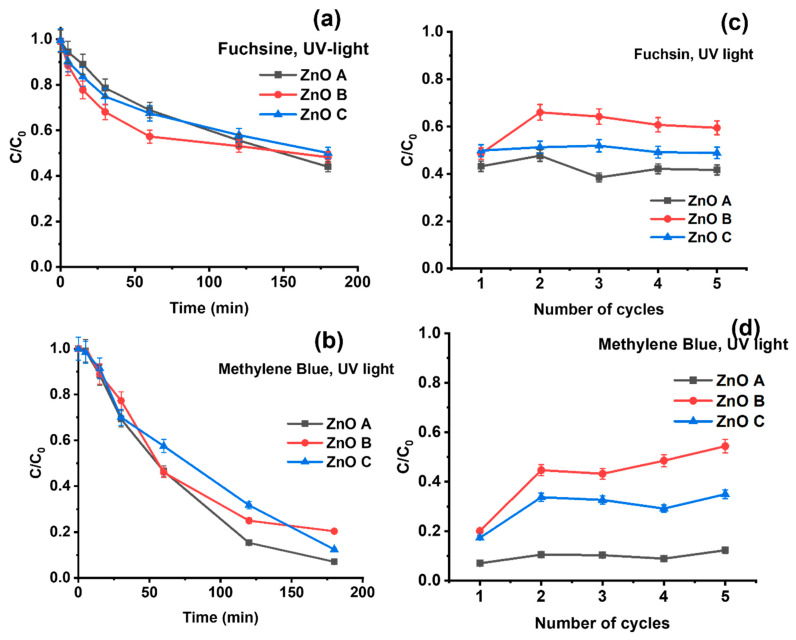
Photocatalytic degradation of a 10 mL solution containing 5 ppm of (**a**) basic fuchsin and of (**b**) methylene blue using 1 mg of ZnO nanoparticles under 365 nm UV light. Photocatalytic degradation of (**c**) basic fuchsin and of (**d**) methylene blue for five cycles of photocatalytic degradation. During each cycle of photocatalysis, the nanoparticles were exposed for 3 h to UV light. The nanoparticles were centrifuged and dried before the next cycle.

The ZnO nanoparticles were applied to the photocatalytic degradation of two dyes: methylene blue and basic fuchsin. Dye degradation or removal is noted as C/C_0_, where C is the concentration of the dye after its removal and C_0_ is the initial concentration. Depending on the experiment, the reduction of C/C_0_ is considered an outcome of both, adsorption of dye molecules, as well as their degradation. In Figure 4a,d, the photocatalytic activity of the three ZnO samples is provided under UV light. For fuchsin removal, the photocatalysts are most efficient in the initial hours with the highest removal rate, as in Figure 5a. This tendency is more prominent for ZnO B-containing nanoparticles with the largest specific surface, displaying the lowest C/C_0_ (i.e., 57.3% at 60 min) or the highest removal rate. However, the removal rate of fuchsin by ZnO B slows down significantly after the first hour. On the other hand, ZnO A has a more constant removal rate, while also having the lowest C/C_0_ after three hours. The different tendencies in the removal rates of fuchsin with ZnO B and ZnO C compared to ZnO A could be a combination of both adsorption and photocatalytic degradation processes. Furthermore, the lower removal rate for ZnO B after the first hour suggests a shielding of the surface of the photocatalysts owing to the adsorption of dye molecules, as explained further in the manuscript.

For methylene blue in Figure 5b, on the other hand, the initial high removal rate for the smallest ZnO nanoparticles (i.e., ZnO B and ZnO C) is absent. During the first 30 min, the dye degradation tendency is similar, i.e., linear for all the three samples. After the initial 30 min, the removal rate decreases slowly but remains higher than for fuchsin. In general, methylene blue is more actively degraded by the ZnO nanoparticles than fuchsin. Once again, ZnO A shows the most efficient degradation reaching removal rates of 90% after 3 h followed by ZnO C with 88% and ZnO B with 80%. 

The reusability of the samples and the sustainability of the photocatalytic process were evaluated for five cycles of dye degradation under 365 nm UV light (Figure 5c,d). For ZnO A, i.e., the sample with the largest crystal size, no significant reduction in photocatalytic activity was observable after five cycles. In addition, ZnO C shows similar removal efficiencies for fuchsin in each cycle of Figure 5c. However, for methylene blue, the removal efficiency decreases after the first utilization, and then shows the same efficiency for four consecutive cycles, as seen in Figure 5d. ZnO C shows a lower efficiency than ZnO A, but a slightly higher efficiency than ZnO B. On the other hand, ZnO B shows a large decline in dye-removal efficiency after the first cycle for both fuchsin and methylene blue. One possible explanation for this decline could be the agglomeration of the nanoparticles due to drying between cycles or the adsorption of dye that degrades the surface of the nanoparticles and, in turn, decreases the photocatalytic degradation efficiency. In addition, the attachment of the dye on to the surface of the nanoparticles can alter the electrostatic charges on their surfaces and promote their agglomeration, which eventually results in lowering the effective specific surface.

Even though the morphology of the nanoparticles plays an important role, other reasons for the decrease in the removal capacity could be the passivation of surface defects via dye adsorption. This surface shielding could explain the lower photocatalytic activities of ZnO B and ZnO C. On the other hand, the presence of sharp edges in ZnO A of Figure 2b, limits shielding because edges cannot be covered, exposing highly active sites on the edges for catalytic reactions. Ni et al. [20], reported that edges are an important part of nanoparticles in the field of catalysis. In their study, they showed that atoms on edge sites are more active due to different chemical environments. They compared their activity with atoms from the facets and showed that atoms on edges promote chemical reactions with higher efficiency. Hejral et al. [37] also investigated the influence of nanoparticle shape in heterogeneous catalysis. They observed that the efficiency of catalytic reactions increases with the number of available edges on the surfaces. 

On the other hand, facets in ZnO nanoparticles can be polar or non-polar. For example, hexagonal nanoparticles contain six polar and two non-polar facets [44]. Polar facets are either Zn- or O-terminated, and harbor the corresponding surface defect, i.e., V_Zn_ or V_O_ [45]. Since the dyes used in this study are cationic, they would most likely attach to the negatively charged V_Zn_. Nevertheless, in aqueous media, hydroxyl groups or oxygen radicals would also be adsorbed on the surface of these nanoparticles at V_O_^++^ that are positively charged, turning their surfaces electronegative and serving as functional groups to attach cationic dye molecules through covalent bonds. These edges are present to some extent in ZnO C, but in high amounts in ZnO A (Figure 2b,d). Therefore, for ZnO A, both V_Zn_ and V_O_ serve as anchoring sites for oxygen radicals and dye molecules. Consequently, despite having the lowest specific surface, ZnO A shows the highest efficiency after five cycles of photocatalytic degradation, without loss in efficiency. In addition, ZnO A has the highest number of volume-related defects, owing to the synthesis conditions that cannot be passivated or shielded, unlike surface defects. However, it is very unlikely that volume-related defects participate in the dye-degradation process. ZnO B harbours surface-related V_O_^++^ and therefore, attracts hydroxyl groups or oxygen radicals. On the other hand, ZnO C that possesses the lowest amount of surface defects due to improved oxidation of the ZnO lattice during its synthesis, consists of shallow donors of Zn_i_ and a negligible amount of surface V_O_^++^. Some of the nanoparticles in ZnO C present sharp edges in Figure 2d, while others present no defined shape. The presence of these sharp edges is probably the explanation of its slightly higher photocatalytic activity compared to ZnO B.

The same experimental conditions, as in the previous section were applied to the study of the photocatalytic degradation of the two dyes under sunlight exposure. It is noteworthy that both fuchsin and methylene blue undergo degradation under sunlight exposure even in the absence of a photocatalyst (control sample), via the process of photobleaching Nevertheless, the presence of ZnO nanoparticles increases the dye-removal rate. For fuchsin, the presence of ZnO nanoparticles increased dye removal by 16–33% depending on the sample, and for methylene blue it increased by 15%.

**Figure 6:**
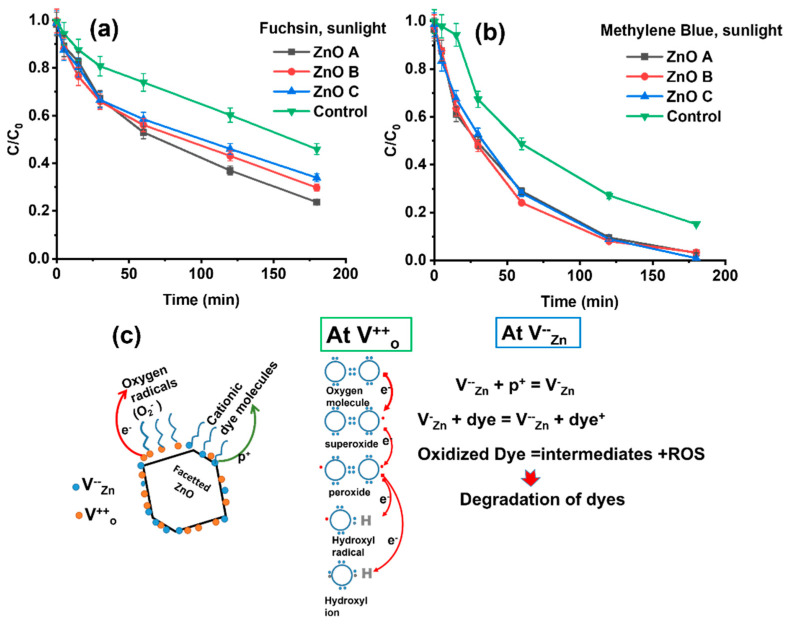
Photocatalytic degradation of a 10 mL solution containing 5 ppm (**a**) basic fuchsin and of (**b**) methylene blue with the three different ZnO samples (1 mg) and photobleaching of the control sample during 3 h of sunlight exposure and (**c**) mechanism of electron transfer from ZnO surface defects on sharp edges and the formation of ROS. The ZnO hexagonal nanoparticle considers only non-polar ZnO surfaces harboring surface defects for the sake of clarity.

In all cases, the degradation follows similar tendencies for the control sample, as well as the samples with the photocatalysts. In the initial stages, a fast decrease in dye concentration is observed during the first 40 min in the presence of the photocatalysts in Figure 6a,b. However, after this initial high removal rate, all curves then follow the same tendency as the control sample, implying that photobleaching becomes the dominant mechanism for dye degradation. Nevertheless, the dye-degradation efficiency is higher by 45% (ZnO A), 33% (ZnO B) and 25% (ZnO C) for fuchsin at the end of 3 h compared to the control sample; the maximum dye-removal efficiency was ~75% for ZnO A. In Figure 5a, ZnO B and ZnO C in fuchsin show lower removal rates under sunlight compared to ZnO A, similar to UV light exposure in Figure 5a. It should be noted that photobleaching in the absence of a photocatalyst is not significant under the UV light of 365 nm, implying that photocatalysis is the only mechanism for the decrease in C/C_0_ under UV light and in the presence of ZnO. This suggests that photons of other wavelengths are responsible for photobleaching under sunlight, which, naturally, are the wavelengths corresponding to the main absorbance peaks of the dye (i.e., 660 nm for methylene blue and 550 nm for basic fuchsin).

For both, fuchsin and methylene blue, the photobleaching depends on the formation of singlet oxygen under oxygen-rich conditions, as photobleaching in de-oxygenated environments is less prominent or even suppressed [46]. In fact, the enhanced photobleaching of methylene blue compared to fuchsin is probably due to the longer lifetimes of the singlet state of the former [22]. This allows a higher number of electrons in the excited singlet-state to undergo intersystem crossing into the triplet state. At this point, they can either follow a type I or a type II photochemical route or de-excite via phosphorescence, along with a change in their spin multiplicity. Type I reactions involve the interaction or electron transfer from the triplet state to biomolecules or organic species. However, like several dyes, triplet states can react with the O_2_ molecule in the ground state or sensitize the production of singlet oxygen in a type II reaction [47]. These oxidizing species, viz., singlet oxygen, superoxides, peroxides, hydroxyl ions and radicals are the main components involved in the dye degradation process as shown in Figure 6c [48,49]. In the presence of electron donors, such as from the conduction band of ZnO on photoexcitation, the dye degradation is enhanced, due to the enhanced production of ROS. In particular, the high number of electrons transferred to oxygen radicals create hydroxyl radicals that finally condense into water.

Photocatalytic studies using ZnO nanorods have shown that the aspect ratio of the nanorods affects the surface defects, which in turn influences the photocatalytic activity of the ZnO nanorods [50]. It has to be considered that nanomaterials with higher aspect ratios tend to have lower quantities of surface defects, unless deliberately induced during the growth process. In contract, nanoparticles of very small sizes harbor surface defects, irrespective of the synthesis condition due to very large specific surfaces. Here, ZnO A with distinct facets harboring V_Zn_ or V_O_, appears to have enhanced photocatalytic degradation properties. Under photoexcitation, electrons are trapped at V_O_^+^/V_O_^++^, and holes are trapped at V_Zn_^−−^ on the surface of the nanoparticle, as shown in Figure 6c. Then, the trapped electron is transferred to the oxygen molecule or radical, creating ROS via a type II mechanism. Simultaneously, holes are trapped at V_Zn_; since the dyes studied are cationic, they have an affinity to the negatively charged V_Zn_ that accepts an electron from the dye molecule, leading to the oxidation of the dye. However, the transfer of the electron from the dye to ZnO nanoparticles can only occur in the singlet state of the dye [51]. The dye oxidation or degradation process is then finalized by the produced ROS. The presence of both types of defects, along with sharp edges appears to be the main reason for a more efficient dye-degradation compared to ZnO B and ZnO C that harbor mainly V_O_^++^.

Since ZnO is a n-type semiconductor, in an aqueous environment, hydroxyl groups and O_2_ radicals attach to the surface of these nanoparticles and produce an upward band-bending [52]. In consequence, a more efficient separation of the photogenerated charges is possible. Another outcome is the suppression of the band-to-band transition due to the increase in the depletion region size around the nanoparticle, whereupon the probability of electron capture at defect sites increases [31]. The upward-band-bending phenomenon is therefore beneficial to the photocatalytic activity.

The photocatalytic dye degradation of methylene blue in Figure 6b is ~20% higher for all the ZnO samples after 3 h of sunlight exposure, and reaches almost 100% degradation for ZnO C. In the present case, the shielding effect due to adsorption of dye molecules is not observed, most probably due to the lack of affinity of the dye to the ZnO nanoparticle surface. Another important effect that increases the rate and efficiency of dye degradation is the wider excitation spectrum of sunlight ranging from the infrared to the UV. While ZnO normally does not absorb visible light due to its large bandgap, the defect states present in nanoparticles enable absorption of photons of visible light around 550 nm in ZnO A (Figure 4a). In fact, a wavelength of 550 nm also corresponds to the most intense emission wavelength of the solar spectrum.

### 3.4. Adsorption Dependent Photocatalytic Degradation

**Figure 7:**
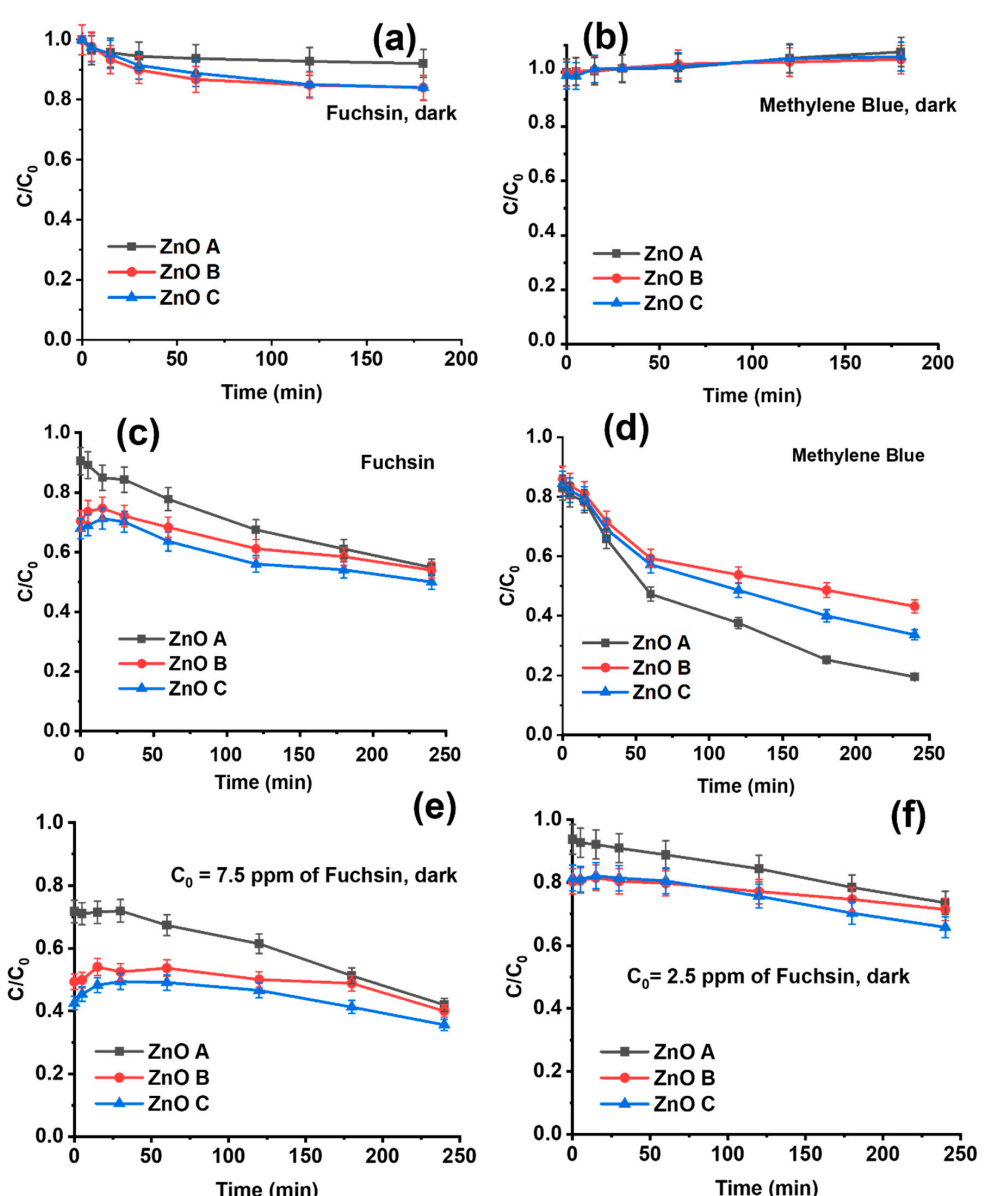
Adsorption study under dark conditions with 10 mL aqueous solution of 5 ppm (**a**) basic fuchsin, and (**b**) methylene blue using 1 mg of the three different ZnO samples for 3 h. Photocatalytic degradation under UV light of a 10 mL aqueous solution containing 5 ppm of dye with 1 mg of ZnO nanoparticles, after reaching adsorption equilibrium (24 h) for (**c**) basic fuchsin, (**d**) methylene blue and for other basic-fuchsin dye concentrations of (**e**) 7.5 ppm and (**f**) 2.5 ppm.

The decrease in fuchsin concentration in the presence of the ZnO nanoparticles under dark conditions highlights a clear adsorption of the dye by ZnO nanoparticles (Figure 6a). Interestingly, ZnO B and ZnO C both appear to have similar high adsorption capacities compared to ZnO A, for which it is significantly lower. This lower adsorption is likely due to the lower surface-to-volume ratio and its smooth crystalline surfaces or facets with certain low-adsorption crystal faces [23,24]. In addition, surface defects can play an important role in the adsorption of dye molecules [18]. Due to its high specific surface, ZnO B will adsorb the highest amount of dye molecules. Regardless of the ZnO morphology and defect states, it appears that methylene blue is not adsorbed by the ZnO nanoparticles under dark conditions for up to 3 h in Figure 7b.

The effect of the preliminary adsorption of dye molecules on the photocatalytic activity of ZnO nanoparticles was investigated. Figure 7c,d show the photocatalytic degradation curves of the three different ZnO samples after 24 h of adsorption in dark conditions. Unlike the photocatalytic degradation curves in Figure 5, C/C_0_ begins at 0.90 for ZnO A, 0.65 for ZnO B and 0.625 for ZnO C, due to the preliminary adsorption of fuchsin in Figure 7c. However, in the case of methylene blue, only 12% of the dye was adsorbed for all the three samples after 24 h, (Figure 7d), with C/C_0_ starting at 0.88.

The higher adsorption of fuchsin molecules on the surface of ZnO B and ZnO C influences their photocatalytic degradation properties significantly. In Figure 7c, an increase in the fuchsin dye concentration is visible immediately under exposure to UV-light, i.e., an increase in C/C_0_, except for ZnO A that showed very low adsorption kinetics in Figure 7a. The reason for the increase in dye concentration under UV exposure is the probable desorption of fuchsin from the surface of ZnO B and ZnO C samples. As basic fuchsin is a cationic molecule, extra surface holes and radicalization of hydroxyl groups under UV exposure can weaken the ionic bonds on the surface, releasing loosely bound molecules. The presence of dye molecule on the surface of the samples acts as a shield against photocatalytic activity, which explains the low removal in the initial stages for ZnO B and ZnO C samples. For methylene blue on the other hand, the desorption was not observable due to low adsorption in Figure 7d. Nevertheless, the overall photocatalytic activity appears less efficient, when photocatalysis follows adsorption.

In Figure 7e,f, we observe that the adsorption capacity does not depend on the dye concentration in the solution. In fact, similar quantities of fuchsin were adsorbed by the different ZnO samples in all three experiments (including the 5 ppm fuchsin concentration in Figure 7c). The adsorption capacity of ZnO A is 0.5 µg/mg and 1.5 µg/mg for both ZnO B and ZnO C samples. Thus, the adsorption capacity is solely determined by the number of active sites available on the ZnO nanoparticle surface, regulated by the synthesis conditions that influence the specific surface and surface defects.

**Figure 8:**
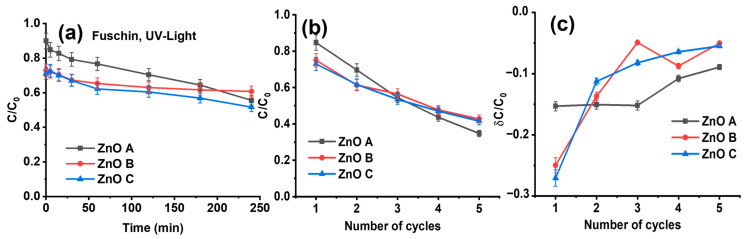
Photocatalytic activity of 1 mg of ZnO nanoparticles in 10 mL aqueous solution of 5 ppm fuchsin under UV light. Prior to photocatalysis, adsorption was carried out for 24 h followed by separating the nanoparticles from the solution, drying them and exposing them for 3 h to UV light. (**a**) The nanoparticles were then returned to the original solution to assess their photocatalytic activity. To check their reusability, the nanoparticles were then isolated and exposed to UV light for 3 h to clean their surfaces, and then returned to the original solution containing fuchsin. The procedure was repeated for four cycles of adsorption and UV-light exposure. (**b**) The cumulative dye removal and (**c**) the C/C_0_ difference for each cycle.

In order to study the possible desorption of the dye molecules from the ZnO nanoparticle surface, as well as the delay in the photocatalytic removal of the dye after adsorption, the ZnO nanoparticles were removed from the fuchsin solution. Then, the ZnO nanoparticles were exposed to UV light for three hours to degrade the fuchsin-dye molecules adsorbed on their surfaces. After this treatment, the fuchsin solution was returned to the Petri dish containing the UV-treated ZnO nanoparticles. Figure 8a shows the photocatalytic activity under UV light of the remaining fuchsin using the UV-treated ZnO nanoparticles. In fact, the UV treatment degraded the adsorbed dye molecules, which consequently suppressed both the initial delay in photocatalytic activity and fuchsin desorption. This clearly suggests that the desorption of dye is responsible for the initial increase in dye concentration in the early stages of photocatalysis. However, both the initial adsorption and the photocatalytic degradation of fuchsin by ZnO B and ZnO C in Figure 8a are significantly lower than in Figure 7, suggesting that dye molecules may have modified the surface of the nanoparticles.

In Figure 8b,c, the experiments consist of several cycles of fuchsin-dye adsorption followed by removal of the adsorbed dye molecules by exposing the ZnO nanoparticles to UV light after isolating them from the dye solution. After a UV-light treatment of 3 h, the treated ZnO nanoparticles were returned to the dye solution for further adsorption. For each cycle, the concentration of fuchsin in the solution decreased as shown in Figure 8b.

Therefore, adsorbed fuchsin is indeed degraded during UV exposure. However, as shown in Figure 8c, the adsorption of fuchsin after the first cycle for ZnO B and ZnO C decreases significantly. For ZnO A, the adsorption is the same for the first three cycles and reduces slightly for cycles 4 and 5. Nevertheless, ZnO A has the highest adsorption capacity after five cycles, among the three samples. In fact, ZnO B and ZnO C exhibited the best adsorption capacity during the first test, but their adsorption capacity decreased after UV treatment, showing a lower reusability. Between these two samples, ZnO C presents the lowest adsorption per cycle that is reduced with every subsequent cycle. This result suggests that the ZnO A, owing to its sharp edges and facets, is more robust and probably exhibits a different surface chemistry than samples ZnO B and ZnO C, which needs to be further investigated. These results suggest that the adsorption of dye molecules on ZnO before photocatalysis tends to give only an initial boost in dye removal. However, these nanoparticles tend to perform less efficiently when adsorption precedes photocatalysis, which, in turn, adversely affects their reusability.

Table 3 summarizes the photocatalytic degradation efficiencies of various nanoparticles, including commercial ZnO nanoparticles against MB. There are no reports of fuchsin degradation with pure ZnO nanoparticles, to the best of our knowledge. In general, all the presented ZnO nanoparticles tend to efficiently degrade MB. Nevertheless, in other studies, the quantity of nanoparticles is higher, i.e., ranging from 15 mg to 4 g compared to the present study, where only 1 mg of nanoparticle was used. The C_0_ of MB in all the solutions was between 5–10 mg/L, similar to the present study. Our nanoparticles appear to efficiently degrade under both UV light and sunlight, with a higher degradation efficiency under sunlight. In particular, ZnO A demonstrates a very high dye degradation efficiency for 1 mg of the photocatalyst after 3 h.

## 4. Conclusions

In this work, we have investigated the photocatalytic activity of ZnO nanoparticles synthesized via three different sol-gel routes, which determined their shape, size and defect states. Solution-based synthesis routes are favorable, owing to the facile handling and controllability of the reaction mechanisms. These nanoparticles were successfully applied to UV-light- and solar-driven photocatalysis of fuchsin and methylene blue. The differences in the morphology due to the synthesis conditions resulted in bandgap states that were the determining factors in the photocatalytic efficiency of these nanoparticles. In fact, the defect states in ZnO create bandgap states, acting as traps that improve charge separation, instrumental in creating ROS. Even though, smaller ZnO nanoparticles with high specific surfaces are extremely efficient photocatalysts; nevertheless, their reusability for several cycles of dye degradation remains limited. On the other hand, ZnO nanoparticles with sharp edges and facets demonstrate a much higher dye-degradation efficiency, reaching nearly 100% in some cases. Clearly, the presence of V_Zn_ along with V_O_ enhances the photocatalytic degradation. In addition, their reusability emphasizes the sustainability of the photocatalytic process. Adsorption preceding photocatalysis appears to be an effective method to initially boost dye removal. However, the dye molecules adhering to the surface of the spherical nanoparticles tend to modify their surface chemistry and prove detrimental to their photocatalytic efficiency and reusability. In that regard, facetted ZnO nanoparticles appear to be good candidates for the removal of more recalcitrant organic pollutants, such as nitrates and pharmaceuticals. Future works will consider a methodology to immobilize these nanoparticles on a support in order to facilitate their recovery after exhaustion for several cycles of dye degradation.

## Figures and Tables

**Table 1:** Parameters obtained from BET for the three samples.

Sample	S_BET_ (m²/g)	C_BET_	$d_{BET=\frac{6000}{S_{BET\times \rho_{ZnO}}}}$ (nm)	d_TEM_ (nm)	Vm (cm^3^/g)	Vp (cm^3^/g)
ZnO A	0.5	74	>50	114	1.2 × 10^−7^	0
ZnO B	72	20	14.8	5	1.66 × 10^−5^	0.23
ZnO C	24	22	44.2	47	5.5 × 10^−6^	0.22

**Table 2:** Description of ZnO nanoparticles.

Sample	Diameter (nm)	Shape	Synthesis Route (Solvent)	Temp (°C)	Reaction Time (h)
ZnO A	80–140	Hexagon, tetrahedron, octahedron with sharp edges and facets.	Non-aqueous (Benzylamine)	300	48
ZnO B	5	Spheres	Non-aqueous (Ethanol)	65	2
ZnO C	27	Spheres, facetted nanoparticles	Aqueous (Ethanol (70%) and water (30%))	60	2

**Table 3:** Comparison of the photocatalytic efficiency of various ZnO nanoparticles against MB.

Photocatalyst	Illumination	C/C_0_	Crystal Size (nm)	Time (min)	C_0_	Mass	Ref
ZnO (commercial Nanograph, Chicago, IL, USA)	UV lamp	0.3	30–50	-	5	4 g	[53]
ZnO (green synthesized)	sunlight	0	200	90	5	2 g	[54]
ZnO (commercial Aldrich)	sunlight	0.03	50	140	10	25 mg	[55]
ZnO (commercial Sigma Aldrich, St. Louis, MO, USA)	Xenon lamp	0.03	50	150	10	25 mg	[56]
ZnO (green synthesized)	UV lamp	0.03	50	100	5	15 mg	[57]
ZnO A (this work)	Sunlight/UV light	0/0.08	100–300	180	5	1 mg	
ZnO B (this work)	Sunlight/UV light	0/0.15	5	180	5	1 mg	
ZnO C (this work)	Sunlight/UV light	0/0.22	50–200	180	5	1 mg	

## Data Availability

Not applicable.

## Supplementary Materials

The following supporting information can be downloaded at: https://www.mdpi.com/article/10.3390/nano13131998/s1, Figure S1: Calibration curves with UV-Vis spectrometer and Lovibond photometer for (a) basic fuchsin at 560 nm and (b) methylene blue at 660 nm; Figure S2: Emission spectrum of the UV-lamp with maximum intensity at 365 nm and an output power; Figure S3: (a) Solar irradiance and (b) UV index during the experiments under direct sunlight. These parameters were almost identical in order to ensure that the photocatalytic degradation was carried out in similar conditions in order to compare the photobleaching, as well as the photocatalytic activity. Methylene blue and fuchsin were manipulated on different days and the red and black curves indicate the solar irradiance and UV index on those days.; Figure S4: Size distribution histograms obtained from TEM images of Figure 2 for ZnO A, ZnO B and ZnO C.
