# Supplementary material for: Revealing the Dependency of Dye Adsorption and Photocatalytic Activity of ZnO Nanoparticles on Their Morphology and Defect States
Source: Nanomaterials (Basel). 2023 Jul 3;13(13):1998. doi: 10.3390/nano13131998 (PMC10343547; doi:10.3390/nano13131998)
Supplement: Supplementary file 1 [file nanomaterials-13-01998-s001.zip › nanomaterials-2490351-supplementary.pdf]

Supporting information

Revealing the dependency of dye adsorption and photocatalytic activity of ZnO nanoparticles on their morphology and defect states.

Yuri Hendrix, Keshav Nagpal, Erwan Rauwel, Elias Estephan and Protima Rauwel

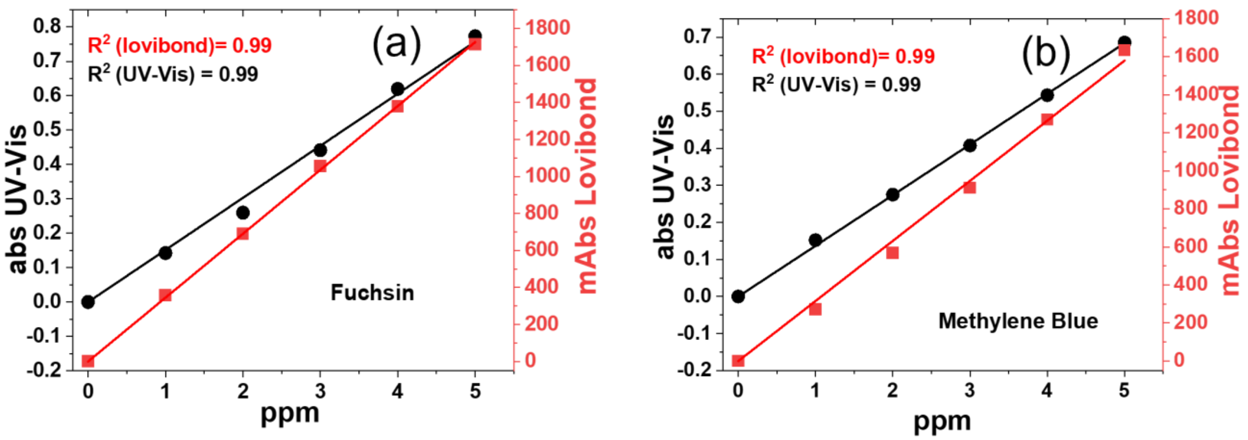

Figure S1: Calibration curves with UV-Vis spectrometer and Lovibond photometer for (a) basic fuchsin at 560 nm and (b) methylene blue at 660 nm.

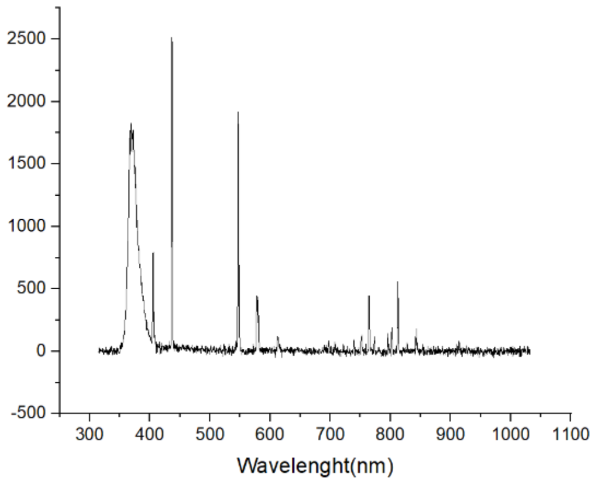

Figure S2: Emission spectrum of the UV-lamp with maximum intensity at 365 nm and an output power of 36W.

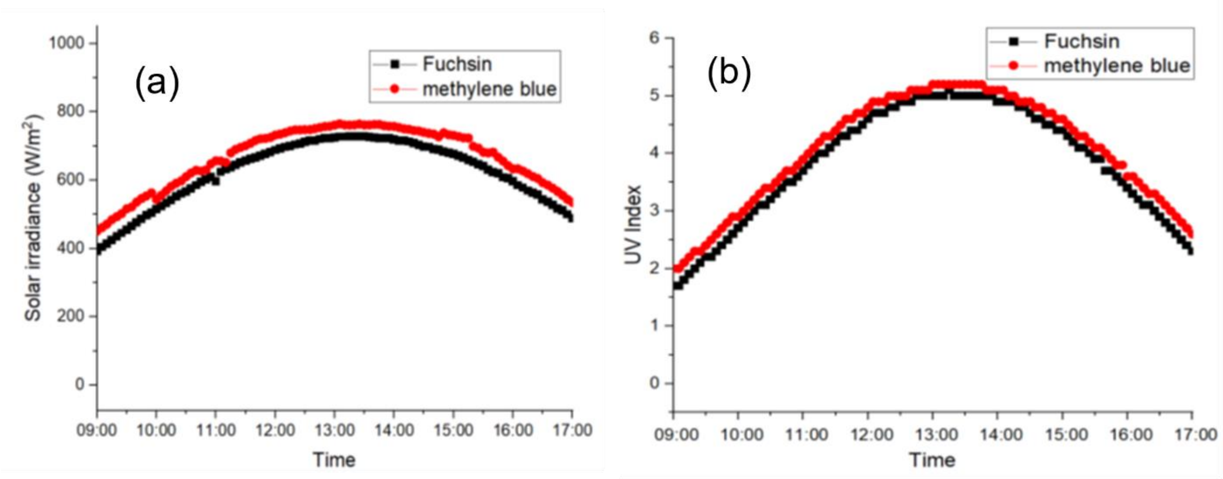

Figure S3: (a)Solar irradiance and (b) UV index during the experiments under direct sunlight. These parameters were almost identical in order to ensure that the photocatalytic degradation was carried out in similar conditions in order to compare the photobleaching, as well as the photocatalytic activity. Methylene blue and Fuchsin were manipulated on different days and the red and black curves indicate the solar irradiance and UV index on those days.

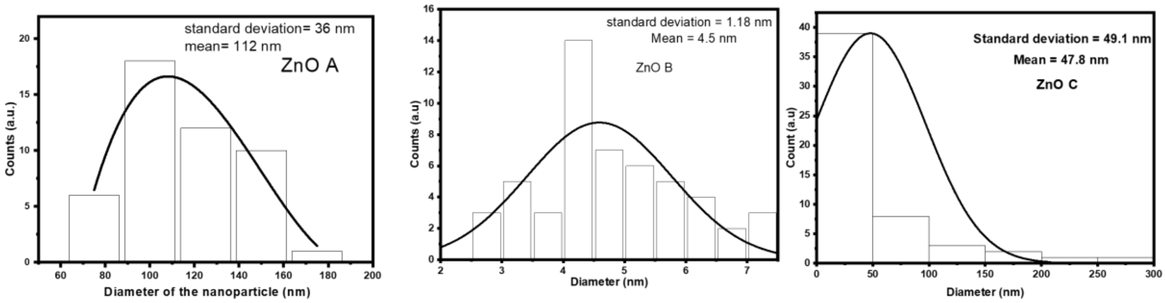

Figure S4: Size distribution histograms obtained from TEM images of Figure 2 for ZnO A, ZnO B and ZnO C.
